# *Candida auris*‒Associated Hospitalizations, United States, 2017–2022

**DOI:** 10.3201/eid2907.230540

**Published:** 2023-07

**Authors:** Kaitlin Benedict, Kaitlin Forsberg, Jeremy A.W. Gold, James Baggs, Meghan Lyman

**Affiliations:** Centers for Disease Control and Prevention, Atlanta, Georgia, USA

**Keywords:** Candida auris, fungi, fungal infections, hospitalizations, epidemiology, United States

## Abstract

Using a large US hospital database, we describe 192 *Candida auris*‒associated hospitalizations during 2017–2022, including 38 (20%) *C. auris* bloodstream infections. Hospitalizations involved extensive concurrent conditions and healthcare use; estimated crude mortality rate was 34%. These findings underscore the continued need for public health surveillance and *C. auris* containment efforts.

*Candida auris* is a highly transmissible and frequently drug-resistant emerging fungal pathogen capable of causing severe infections. *C. auris* can colonize skin, leading to infection and transmission in healthcare settings. In the United States, reported clinical cases increased by 95% during 2020–2021 (*1*). US data on *C. auris* come primarily from case series and outbreak investigations and are geographically limited, and national surveillance data lack detail on patients’ underlying conditions, healthcare use, and outcomes. Therefore, we used a large healthcare services database to describe features of hospitalized patients with *C. auris* infection or colonization.

The PINC-A1 Healthcare Database (PHD) (https://offers.premierinc.com/rs/381-NBB-525/images/PINC_AI_Healthcare_Data_White_Paper.pdf) is a hospital-based all-payer database that contains healthcare use, financial, and pharmacy data from >1,000 US hospitals. Laboratory data are available from ≈25% of those hospitals. We identified all hospitalizations with a culture positive for *C. auris* from any specimen type during 2017–2022. We used diagnosis codes from the International Classification of Diseases 10th Revision, Clinical Modification, to identify underling conditions and complications (Appendix Table) and billing data to identify medical devices. We assessed features of *C. auris* hospitalizations and compared those with versus those without bloodstream infection (BSI) by using χ^2^, Fisher exact, and Wilcoxon tests (α = 0.05).

A total of 192 *C. auris* hospitalizations (38 [20%] with BSI) occurred in 42 hospitals. *C. auris* hospitalizations primarily occurred among older adults (median age 68 years [range 21–89 years]), male patients (54%), and non-Hispanic White patients (60%). Non-Hispanic Black patients more frequently had BSI than did other races/ethnicities (39% vs. 29%; p = 0.022) (Table). The first positive *C. auris* specimen was collected within 2 days of admission for 63% of bloodstream and 48% of nonbloodstream *C. auris* hospitalizations. Among hospitalizations with bloodstream *C. auris*, 58% also had another positive specimen type. Among hospitalizations without bloodstream *C. auris*, the most common positive specimen types were axilla (38%) and urine (34%).

**Table:** Characteristics of hospitalizations involving *Candida auris,* United States, 2017–2022*

Characteristics	Total, n = 192	Bloodstream infection,† n = 38	Nonbloodstream infection,‡§ n = 154	p value
Mean, median age, y (range)	66.0, 68.0 (21–89)	61.8, 66.5 (32–86)	67.0, 68.0 (21–89)	0.042
Age category, y				0.264
<45	22 (11.5)	§	§	
45–64	52 (27.1)	11 (28.9)	41 (26.6)	
>65	118 (61.5)	20 (52.6)	98 (63.6)	
Sex				0.193
M	104 (54.2)	17 (44.7)	87 (56.5)	
F	88 (45.8)	21 (55.3)	67 (43.5)	
Race/ethnicity, n = 186				0.022
Non-Hispanic Black	45 (24.2)	14 (38.9)	31 (20.7)	
All other races/ethnicities¶	141 (75.8)	22 (61.1)	119 (79.3)	
Payer				0.483
Medicare	128 (66.7)	25 (65.8)	103 (66.9)	
Medicaid	35 (18.2)	§	§	
Private health insurance	20 (10.4)	§	§	
Other	§	§	§	
Clinical features				
Mean, median time from admission to first positive *C. auris* specimen collection, d (range)	8.6, 2.0 (1–187)	8.7, 1.0 (1–53)	8.6, 3.0 (1–187)	0.997
Within 2 d	98 (51.0)	24 (63.2)	74 (48.1)	
>2 d	94 (49.0)	14 (36.8)	80 (51.9)	
Systemic antifungal use on or after first positive *C. auris* culture				
Echinocandin	68 (35.4)	29 (76.3)	39 (25.3)	<0.001
Fluconazole	20 (10.4)	§	§	0.081
Amphotericin B	§	§	§	0.016
Underlying conditions and complications				
Sepsis	123 (64.1)	25 (65.8)	98 (63.6)	0.804
Diabetes	106 (55.2)	20 (52.6)	86 (55.8)	0.721
Chronic kidney disease	85 (44.3)	17 (44.7)	68 (44.2)	0.949
Pneumonia	83 (43.2)	17 (44.7)	66 (42.9)	0.834
Chronic respiratory failure	61 (31.8)	15 (39.5)	46 (29.9)	0.255
Liver disease	29 (15.1)	§	§	0.099
COVID-19	24 (12.5)	§	§	0.891
Solid organ malignancy	21 (10.9)	§	§	0.502
Hematologic malignancy	§	§	§	1.000
HIV	§	§	§	0.486
Neutropenia	§	§	§	1.000
Transplant and complications	§	§	§	0.585
Medical devices				
Central venous catheter	111 (57.8)	29 (76.3)	82 (53.2)	0.010
Mechanical ventilation	83 (43.2)	18 (47.4)	65 (42.2)	0.565
Tracheostomy	29 (15.1)	11 (28.9)	18 (11.7)	0.008
Feeding tube	16 (8.3)	§	§	0.913
Urinary catheter	17 (8.9)	§	§	0.297
Total parenteral nutrition	17 (8.9)	§	10 (6.5)	0.021
Outcomes				
Mean, median length of hospitalization, d (range)	18.6, 13 (1–209)	22.5, 16.5 (1–65)	17.6, 12.0 (1–209)	0.212
Intensive care unit stay	145 (75.5)	30 (78.9)	115 (74.7)	0.583
Discharge status				0.046
In-hospital death or discharged to hospice#	65 (33.9)	18 (47.4)	47 (30.5)	0.123
Discharged to skilled nursing facility	53 (27.6)	§	§	
Discharged to long-term care acute hospital	28 (14.6)	§	§	
Other	46 (24.0)	§	§	

*Values are no. (%) patients except as indicated. Data were from the PINC-A1 Healthcare Database (PHD) (https://offers.premierinc.com/rs/381-NBB-525/images/PINC_AI_Healthcare_Data_White_Paper.pdf). Most *C. auris* hospitalizations were in the South (76.6%) or the Midwest (21.4%) regions; <10 *C. auris* hospitalizations were in the Northeast or the West regions; >95% of hospitals were in urban locations. A total of 35.4% of *C. auris* hospitalizations were from 2022, 51.0% were from 2021, and 13.5% were from 2017–2020. We found 0 *C. auris* hospitalizations for 2012–2016. †A total of 22 (57.9%) patients also had *C. auris* isolated from another specimen type; among those, 81.8% had both specimens collected on the same day, and the remainder had the bloodstream specimen collected first. ‡Specimen types: 59 axilla, 53 urine, 18 wound, 13 respiratory, 21 other or unspecified. §Nonzero number <10 or cell that would enable calculation of another cell <10. ¶Includes 112 (60.2%) non-Hispanic White and 21 (11.3%) Hispanic. #Forty (20.8%) in-hospital deaths and 25 (13.0%) discharged to hospice.

Underlying conditions and complications were similar for patients with bloodstream and nonbloodstream *C. auris* and most commonly were sepsis (64%), diabetes (55%), chronic kidney disease (44%), and pneumonia (43%). Compared with nonbloodstream *C. auris*, bloodstream *C. auris* hospitalizations more frequently involved central venous catheters (CVC) (76% vs. 53%; p = 0.010) and tracheostomies (29% vs. 12%; p = 0.008). Echinocandin use was more frequent for bloodstream (76%) versus nonbloodstream (25%) hospitalizations; median time from first positive culture to echinocandin use was 2 days (interquartile range 1–3 days).

Most (75.5%) hospitalizations involved an intensive care unit stay; mechanical ventilation was used in 43% of hospitalizations. Median hospitalization length was 13 days (range, 1–209 days). In-hospital mortality rate was 21%; discharge locations included hospice (13%), skilled nursing facility (28%), and long-term acute care (15%). Estimated crude mortality rates were 47% for bloodstream *C. auris* vs. 31% for nonbloodstream.

This analysis of a large convenience sample of *C. auris*‒associated hospitalizations provides information about clinical features that are currently unavailable through national public health surveillance. Our results support smaller previous investigations showing that infection and colonization with *C. auris* occurs most commonly in patients with complex medical conditions (*2**–**5*). The proportion of *C. auris* cases involving BSI (20%) was comparable to the 9%–28% BSI rate among clinical and screening cases found in previous state-specific studies (*2*,*6*). Including in-hospital deaths and discharges to hospice, the overall estimated crude mortality rate of 34% (47% for BSI) was similar to the 30-day mortality rate from a previous study in New York (27% overall and 39% for BSI) (*2*).

Consistent with candidemia treatment guidelines, most BSI hospitalizations involved echinocandin use, and treatment lag was typical (*7*). Hospitalizations involving *C. auris* BSI were associated with non-Hispanic Black race, similar to those for non–*C.* *auris* candidemia (*8*). The association between CVC use and *C. auris* BSI is not surprising, given that CVC use is a well-documented risk factor for candidemia and is common among patients with *C. auris*, because extensive healthcare exposure, intensive care unit stays, and use of medical devices are key factors in *C. auris* acquisition (*2**,**9*). Many BSI patients were probably admitted with *C. auris,* based on first positive blood specimens occurring soon after admission, similar to finding from a New York case series (*3*). However, we could not assess previous healthcare exposures and prehospitalization laboratory data, which are major considerations because patients with *C. auris* usually acquire it in healthcare settings and can remain colonized for months (*9*).

Other limitations of our study include lack of antifungal susceptibility testing data, possible underdetection of cases caused by potential incompleteness of 2022 data, and underrepresentation of the West and the Northeast regions in PHD laboratory data (*10*), which is particularly relevant because those regions report high case counts (https://www.cdc.gov/fungal/candida-auris/tracking-c-auris.html). PHD laboratory data also underrepresent rural, smaller hospitals (*10*), potentially further biasing this convenience sample of *C. auris* cases. In conclusion, this analysis of hospitalization data supports previous targeted reports and demonstrates a need for strengthened national surveillance and further studies to identify risk factors for *C. auris* infection and colonization.

Appendix Additional information on *Candida auris*‒associated hospitalizations, United States, 2017–2022.
