# Supplementary material for: Candida auris‒Associated Hospitalizations, United States, 2017–2022
Source: Emerg Infect Dis. 2023 Jul;29(7):1485–7. doi: 10.3201/eid2907.230540 (PMC10310363; doi:10.3201/eid2907.230540)
Supplement: Appendix — Additional information on Candida auris‒associated hospitalizations, United States, 2017–2022. [file 23-0540-Techapp-s1.pdf]

Article DOI: https://doi.org/10.3201/eid2907.230540

EID cannot ensure accessibility for Supplemental Materials supplied by authors. Readers who have difficulty accessing supplementary content should contact the authors for assistance.

Candida auris–Associated Hospitalizations, United States, 2017–2022

Appendix

Appendix Table. ICD-10-CM codes used to define underlying conditions and complications

Description	ICD-10-CM code(s)
Chronic kidney disease	N18
COVID-19	U07.1, B97.29
Diabetes	E08–E13
Hematologic malignancy	C81–C86, C88, C90–C96
HIV	B20, Z21
Liver disease	K70–K77
Neutropenia	D70
Pneumonia	J12–J18
Chronic respiratory failure	J96.1, J96.2
Sepsis	A40–A41
Solid organ malignancy	C00–C80 (excluding C44)
Transplant and complications	T86, Z94 (excluding Z94.7), Z95.2, Z95.3
